# Comparison of Zn, Cu, and Fe Content in Hair and Serum in Alopecia Areata Patients with Normal Group

**DOI:** 10.1155/2014/784863

**Published:** 2014-08-27

**Authors:** Ladan Dastgheib, Zohreh Mostafavi-pour, Ahmad Adnan Abdorazagh, Zahra Khoshdel, Maryam Sadat Sadati, Iman Ahrari, Sajjad Ahrari, Mahsa Ghavipisheh

**Affiliations:** ^1^Molecular Dermatology Research Center, Dermatology Department, Shiraz University of Medical Sciences, Shiraz, Iran; ^2^Maternal-Fetal Medicine Research Center, Hafez Hospital, Shiraz University of Medical Sciences, Shiraz, Iran; ^3^Recombinant Protein Laboratory, Department of Biochemistry, Shiraz University of Medical Sciences, Shiraz, Iran; ^4^Student Research Committee, Shiraz University of Medical Sciences, Shiraz, Iran; ^5^Department of Biology, Shiraz University, Shiraz, Iran; ^6^Student Research Committee, Fasa University of Medical Sciences, Fasa, Iran

## Abstract

*Background*. Alopecia areata (AA) is an autoimmune condition, in which hair is lost from some areas of the body. Though its etiopathogenesis is not fully understood, there are claims that imbalance of trace elements may trigger the onset of AA, by distorting immune functions. In this study, we tried to investigate the relationship between AA and iron, zinc, and copper levels of serum and hair. *Materials and Methods*. Sixteen female patients with AA (14–40 years old) and 27 healthy female controls were enrolled in this study. Serum and hair level of iron, zinc, and copper were measured by flame emission spectroscopy. The resulting data was analyzed with SPSS15. *Results*. We did not detect a significant difference in the serum and hair level of iron, zinc, and copper between patients and controls. There was a significant correlation between serum and hair level of iron (*r* = 0.504,  *P* = 0.001), zinc (*r* = 0.684,  *P* = 0.0001), and copper (*r* = 0.759, *P* = 0.0001) in patients and controls. *Discussion and Conclusion*. According to this study, there was no statistically significant difference between trace elements among AA patients and controls. So the trace elements level in hair and serum may not be relevant to the immunologic dysfunction that exists in AA patients.

## 1. Introduction

Alopecia areata (AA) is a recurrent, nonscarring hair loss, affecting any hair-bearing area. Its incidence is 1-2%. AA is considered to be a T-cell-mediated autoimmunity occurring in genetically predisposed individuals [1]. In addition to immune function disturbance, genetic and environmental factors play a role [2]. Also, perifollicular vasculature and nerves, viruses, alterations in trace elements [3], and endocrine and thyroid abnormality [4] have been hypothesized. Complex interactions between predisposing genetic and environmental factors likely contribute to the induction of immune-mediated responses in AA [4].

Clinically, AA has many different patterns. The characteristic lesion is a flat alopetic plaque with normal skin color, involving the scalp or any other region of the body [5]. There are claims that imbalance of trace elements may trigger the onset of AA.

Reports have been published on oral zinc sulfate therapy with encouraging results for some cases of AA [6, 7]. It has been reported that some AA patients have zinc and some other trace element deficiencies [8, 9].

Trace elements are essential cofactors for multiple enzymes and have a role in important functional activities within the hair follicle. Further, zinc accelerates hair follicle recovery and is a potent inhibitor of hair follicle regression [10]. Iron and zinc are the well-known trace elements that are associated with hair shedding [10, 11]. In spite of the fact that several studies were done on the effect of trace elements in AA, a definite result was not obtained. Therefore, in this study, we tried to investigate the relationship between AA and some trace elements in our population. At the same time, we are going to evaluate the correlation between serum and hair contents of these trace elements.

## 2. Materials and Methods

Sixteen female patients and 27 female healthy individuals were enrolled in this case control study. The patients had localized hair loss and were clinically diagnosed as AA with typical lesion. The control group was selected from healthy individuals who did not use any minerals in the last 6 months and did not have any history of hair loss. Patients with history of anemia, thalassemia, and metabolic disorders as well as patients who dyed their hair were excluded from the study. Case and control groups were matched for age. All patients were informed about the study, and their participation was voluntary.

After taking demographic data, hair analysis was made on approximately 0.5 g of hair samples obtained from the scalp of the cases and controls. The wet digestion involved the addition to the sample of 6 mL of nitric acid, which was allowed to react slowly at room temperature to prevent excessive foaming. Five milliliter of blood was obtained for determination of the level of trace elements in the serum.

After that, when warming the nitric acid digest, 1 mL of perchloric acid was added, and the digestion continued on a hot plate at about 200°C until dense white fumes of perchloric acid were evolved. At this point, the mixture was water-clear and less than 1 mL of solution remained. Each sample was transferred to a 5 mL volumetric flask and diluted to volume for copper, iron, and zinc.

Working standards for each element were prepared by dilution of 1000 micro g/mL standard solution. Dilutions were made with distilled water.

### 2.1. Determination of Serum Copper with Flame Emission Method

For determination of serum copper (Cu), the samples were diluted 1 : 4 with deionized water for flame emission spectroscopy methods. Cu standards were prepared by diluting the copper stock standard solution with deionized water. Hollow cathode lamp for Cu was used.

### 2.2. Determination of Serum Zinc with Flame Emission Method

For determination of serum zinc, the samples were diluted 1 : 5 with deionized water. Zinc standards were prepared by diluting the zinc stock standard solution with deionized water. Hollow cathode lamp for Zn was used.

### 2.3. Determination of Serum Iron with Flame Emission Method

To determine total serum iron (Fe), samples were diluted 1 : 2 with a 10% (v/v) trichloroacetic acid (TCA) solution and then centrifuged. This procedure precipitated the serum protein and removed approximately 95% of any hemoglobin iron. Hollow cathode lamp for Fe was used. Analyses were performed using a PerkinElmer model 300 atomic absorption spectrophotometer.

Data were analyzed under supervision of a statistician specialist with SPSS 15. The following statistical methods were used: Fisher's exact test, paired-sample* t* test and two-tailed test of significance. *P* value 0.05 and less was considered as significant. Correlation analysis was carried out using Pearson's correlation and regression analysis.

## 3. Results

Sixteen female patients and 27 female healthy individuals were enrolled in this case control study. Mean age of patients was 26.63 (±8.53) years and controls 25.07 (±5.01) years, which was not statistically significant. Only history of AA in patients and their family and occupation were different in cases and controls.

History of DM (diabetic mellitus), TY (thyroid disease), and AU (other autoimmune diseases) was not different in patients, their families, and controls. Mean duration of disease among the patients was 23.69 (±41.55) months.

The demographic data and associated disease of patients and control are illustrated in Table 1.

We did not detect a significant difference in the serum level and hair level of iron, zinc, and copper between patients and controls (Table 2).

As it is evident from Tables 3 and 4 there was no correlation found between trace element content of hair and serum when compared two by two; the *P* values are not significant (>0.05) and Pearson correlation coefficient is very small, almost near zero. The only interesting exception was a negative relation between serum iron and zinc level evident by *P* = 0.04 (Table 4).

There was a significant correlation between serum and hair level of iron (*r* = 0.524, *P* = 0.001), zinc (*r* = 0.684, *P* = 0.0001), and copper (*r* = 0.759, *P* = 0.0001) in patients and controls.

**Figure 1:**
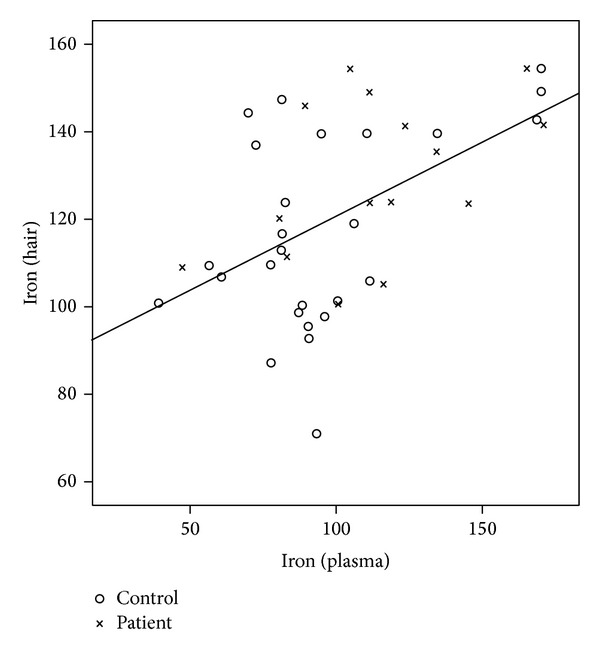
Correlation between plasma and hair iron level in patients and controls.

**Figure 2:**
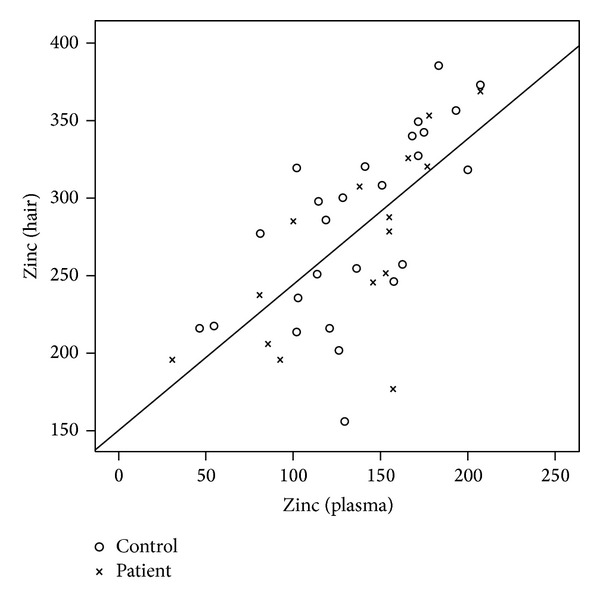
Correlation between plasma and hair zinc level in patients and controls.

**Figure 3:**
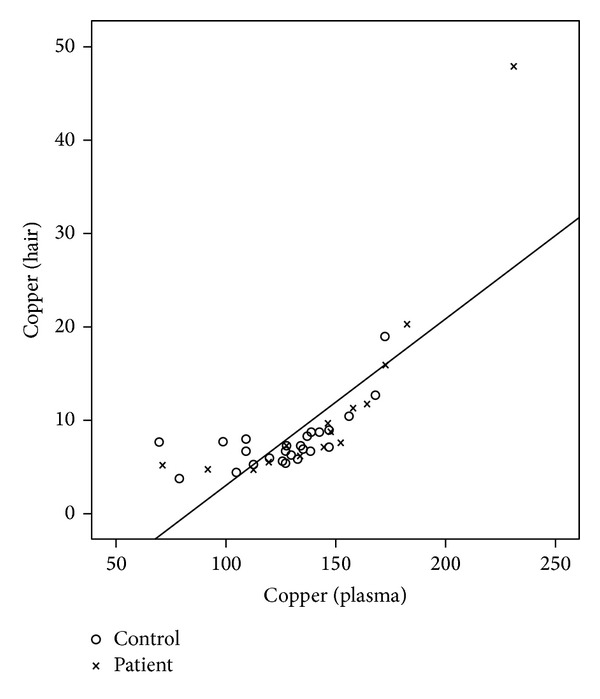
Correlation between plasma and hair copper level in patients and controls.

This is also shown in Figures 1, 2, and 3 with a linear configuration.

## 4. Discussion

Our results showed that the level of zinc, iron, and copper was not significantly different in our patients compared to that of controls.

In review of the literature, there are several investigations that studied the mineral and nutritional conditions in patients with hair loss, especially AA.

Naginiene et al. [4] found a lower level of zinc in blood and urine of children with alopecia and increased levels of copper and chromium concentrations in their hair compared to healthy individuals [4]. Bruske and Salfeld [10] interpreted the statistical association of blood and serum levels of zinc, magnesium, and copper in patients with many dermatological disorders including AA. After comparing with healthy people they did not find any changes in serum levels of zinc and copper but found a significantly higher level of magnesium [10]. Kantor et al. [11] found that the mean ferritin level in patients with androgenetic alopecia and AA was statistically significantly lower than in normal individuals without hair loss [11]. The trace element concentrations of Se, Rb, Zn, Fe, Co, Cs, Mg, Ca, F, Cu, Cr, and Ag in serum and of Se, Rb, Zn, Fe, Co, and Cs in red cells of Finnish alopecia patients were determined in Mussalo-Rauhama study [3]. In addition, the Cu and Zn content in 24 h urine and Cu, Zn, Cd, Cr, and Se concentrations in the hair of these patients were studied. No differences in element concentrations of the samples mentioned above could be found as compared to those of the normal healthy individuals. In addition, there was no tendency of excesses or deficiencies of elements analyzed in the samples. Statistically significant difference was found between the copper content of serum in AA and alopecia universalis patients and also between the copper content of serum in AA plus alopecia totalis and alopecia universalis patients [3].

Although immunologic processes and hereditary factors are suggested to play an important role in AA, the specific etiology is unclear. Iron deficiency has been suggested to play a role, but its effect is controversial. Esfandiarpour et al. [12] found a higher mean level of serum iron and ferritin and a lower mean level of TIBC in AA patients compared to the control subjects, but the differences did not reach significance [12]. The study of Park et al. [13] suggested that zinc supplementation could become an adjuvant therapy for AA patients with a low serum zinc level and for whom the traditional therapeutic methods have been unsuccessful [13]. Bhat et al. [14] showed in their study that copper and magnesium levels are not altered in AA, but they mentioned that the decreased level of zinc found in their study may merit further investigation of the relationship [14].

As mentioned, AA is thought to be an autoimmune disorder, in which the body attacks its own hair follicles and suppresses or stops hair growth. There is evidence that T cell lymphocytes cluster around these follicles, causing inflammation and subsequent hair loss. It is now found that nutritional deficiency of zinc and the other trace elements in human populations may distort immune function. As it has been noted, there are controversial data from different studies. The varied results of the levels of magnesium, copper, and zinc in various studies can be explained on the basis of sample size, methodology, and population variations. Our study, however, suggests that low level of trace elements may not have an important role in immunologic dysfunction in AA patients.

Although, in Bhat et al. study [14], they showed a significant difference in serum zinc levels in AA patients, it was mentioned that these results were seen in AA patients with extensive, prolonged, and resistant to treatments cases. However, most patients included in our study were mild to moderate cases. AA patients who were totalis and universalis were not enrolled in our study.

According to inconclusive data, it has been shown that the empiric therapy with mineral supplements was not very effective in the majority of AA patients. It is prudent to check the level of trace elements serum level in AA patients, and if low serum levels of trace elements are detected, it is advised to prescribe mineral supplements as an adjuvant therapy.

At the same time, we intended to evaluate the level of trace elements in the hair. The results showed a significant correlation between the level of iron and zinc in the serum and the hair. A stronger relation proved itself between the level of copper in the hair and in the serum.

Among the two groups, the results of the iron level showed a stronger relation in the normal healthy controls than in patients, while for the zinc level, results showed a strong relation in both controls and patients.

## 5. Conclusion

In conclusion, trace elements cannot be considered as a direct etiologic factor in the pathogenesis of alopecia areata and not all AA patients may benefit from receiving nutritional supplements. We rather suggest checking trace elements level in these patients and adding supplement in those with documented deficiency as an adjuvant to the usual treatment.

In addition, our results showed that the measurement of hair zinc, iron, and copper level may give us an approximate estimate to its level in serum.

Our study had several limitations. First of all, we could not include male patients as 0.5–1 g sampling from male scalp would cause significant cosmetic defect. Secondly, we could not consider severe AA cases such as totalis and universalis, as they yielded no hair samples and, hence, limited the number of our patients.

## Figures and Tables

**Table 1:** Baseline demographics and associated diseases among patients and controls.

Parameter		Case (*n* = 16)	Control (*n* = 27)	*P* value
Age (mean ± SD) (years)		26.63 ± 8.53	25.07 ± 5.01	0.515

Disease duration (months)		23.69 ± 41.55		

Occupation	Student House wife Employee	5 (31.3) 9 (56.3) 2 (12.5)	13 (48.1) 2 (7.4) 12 (44.4)	0.001

AA	No Yes	9 (56.3) 7 (43.8)	27 (100) 0 (0)	0.0001

DM	No Yes	15 (93.8) 1 (6.3)	27 (100) 0 (0)	0.372

TY	No Yes	15 (93.8) 1 (6.3)	26 (96.3) 1 (3.7)	0.611

AU	No Yes	16 (100) 0 (0)	27 (100) 0 (0)	1

AA in family	No Yes	13 (81.3) 3 (18.8)	27 (100) 0 (0)	0.045

DM in family	No Yes	12 (75) 4 (25)	20 (74.1) 7 (25.9)	0.621

TY in family	No Yes	13 (81.3) 3 (18.8)	26 (96.3) 1 (3.7)	0.137

AU in family	No Yes	15 (93.8) 1 (6.3)	27 (100) 0 (0)	0.327

Pitting nail	No Yes	15 (93.8) 1 (6.3)	27 (100) 0 (0)	0.327

AU: other autoimmune diseases, AA: alopecia areata, DM: diabetes mellitus, TY: thyroid disease.

**Table 2:** Serum and hair level of trace elements in patients and controls.

Parameter	Case (*n* = 16)	Control (*n* = 27)	*P* value∗
Serum Fe (*µ*g/dL)	108 ± 36	96.01 ± 33	0.251
Hair Fe (*µ*g/g)	128 ± 18	117.84 ± 22	0.121
Serum Zn (*µ*g/dL)	134 ± 46	136.76 ± 41	0.877
Hair Zn (*µ*g/g)	270 ± 58	279.35 ± 61	0.65
Serum Cu (*µ*g/dL)	143 ± 38	128.32 ± 23	0.12
Hair Cu (*µ*g/g)	52 ± 62	67.59 ± 59	0.441

∗Two-sample *t*-test.

**Table 3:** Correlation between trace elements measured in hair.

		Zinc (hair)	Iron (hair)	Copper (hair)
Zinc (hair)	Pearson correlation	1	.100	.192
	Sig. (2-tailed)		.517	.211
	*N*	44	44	44

Iron (hair)	Pearson correlation	.100	1	.177
	Sig. (2-tailed)	.517		.251
	*N*	44	44	44

Copper (hair)	Pearson correlation	.192	.177	1
	Sig. (2-tailed)	.211	.251	
	*N*	44	44	44

**Table 4:** Correlation between serum levels of trace elements.

		Zinc (plasma)	Iron (plasma)	Copper (plasma)
Zinc (plasma)	Pearson correlation	1	−.319∗	.104
	Sig. (2-tailed)		.042	.516
	*N*	41	41	41

Iron (plasma)	Pearson correlation	−.319∗	1	.142
	Sig. (2-tailed)	.042		.377
	*N*	41	41	41

Copper (plasma)	Pearson correlation	.104	.142	1
	Sig. (2-tailed)	.516	.377	
	*N*	41	41	41

∗Correlation is significant at the 0.05 level (2-tailed).
